# Ontology-based prompt tuning for news article summarization

**DOI:** 10.3389/frai.2025.1520144

**Published:** 2025-02-11

**Authors:** A. R. S. Silva, Y. H. P. P. Priyadarshana

**Affiliations:** Informatics Institute of Technology, Colombo, Sri Lanka

**Keywords:** knowledge representation, natural language processing (NLP), ontology, prompt tuning, text summarization

## Abstract

Ontology-based prompt tuning and abstractive text summarization techniques represent an advanced approach to enhancing the quality and contextual relevance of news article summaries. Despite the progress in natural language processing (NLP) and machine learning, existing methods often rely on extractive summarization, which lacks the ability to generate coherent and contextually rich summaries. Moreover, these approaches rarely integrate domain-specific knowledge, resulting in generic and sometimes inaccurate summaries. In this study, we propose a novel framework, which combines ontology-based prompt tuning with abstractive text summarization to address these limitations. By leveraging ontological knowledge, our model fine-tunes the summarization process, ensuring that the generated summaries are not only accurate but also contextually relevant to the domain. This integration allows for a more nuanced understanding of the text, enabling the generation of summaries that better capture the essence of the news articles. Our evaluation results demonstrate significant improvements over state-of-the-art methods such as BART, BERT, and GPT-3.5. The results show that the proposed architecture achieved a 5.1% higher ROUGE-1 score and a 9.8% improvement in ROUGE-L compared to baseline models. Additionally, our model showed significance in F1, precision, and recall metrics, with major improvements of 6.7, 3.9, and 4.8%, respectively. These results underscore the effectiveness of integrating ontological insights into the prompt tuning process, offering a robust solution for generating high-quality, domain-specific news summaries.

## 1 Introduction

The explosion of information in the digital age, particularly in the realm of news media, presents a significant challenge: the sheer volume of content makes it increasingly difficult for readers to efficiently identify and digest pertinent information (Steen and Markert, 2024). Traditional text summarization methods, as observed in recent studies (Islam et al., 2021; Schick and Schütze, 2021; Liang et al., 2023; Raundale and Shekhar, 2021), often fail to capture the nuanced context and maintain factual accuracy, resulting in summaries that are incomplete, overly general, or inaccurate. This underscores the necessity for advanced summarization techniques that can generate concise yet informative summaries, especially for news articles where timeliness and relevance are critical (Singh et al., 2021).

This study proposes a novel approach to news article summarization that leverages prompt tuning and ontology integration to address the limitations of current summarization methods. Ontology, a structured representation of knowledge within a specific domain, has shown potential in enhancing various applications in information science and artificial intelligence, such as semantic web, knowledge management, and natural language processing (NLP) (Grimm et al., 2011; Elsayed, 2023). However, the integration of ontologies into domain-specific prompt generation for text summarization remains largely unexplored due to the perceived complexity and difficulty of the process (Kumar and Solanki, 2023).

Prompt tuning, a critical process in refining text summarization systems, involves generating and iteratively refining prompts to guide the summarization process towards producing relevant and coherent summaries. Despite its significance, prompt tuning presents challenges in crafting prompts that effectively capture complex textual content while maintaining brevity and relevance (Zhang et al., 2017). The combination of prompt tuning with ontology integration in the context of abstractive text summarization offers a promising avenue for improving the quality and contextuality of news article summaries.

Abstractive text summarization, unlike extractive methods, generates novel sentences that encapsulate the essence of the input text, offering more concise and coherent summaries. This method, when coupled with ontology-based prompt tuning, can significantly enhance the relevance and accuracy of news article summaries, ensuring that the generated content is both contextually rich and factually accurate. Despite the potential of abstractive summarization, challenges such as ensuring factual accuracy, coherence, and fluency remain (Ma et al., 2023).

The motivation behind this research lies in addressing the shortcomings of existing summarization techniques by exploring the integration of ontologies, prompt tuning, and abstractive summarization methods. By automating the summarization process and leveraging domain-specific knowledge, this study aims to improve the efficiency of information access and knowledge dissemination in the domain of news article summarization. This research is particularly relevant in the context of sports news, where the dynamic nature of the content demands continuous adaptation of summarization models to evolving contexts (Hermida, 2014).

The remainder of this paper is organized as follows: Section 2 provides the background context for this study, including an overview of text summarization techniques, ontology integration, prompt generation and tuning, as well as the research gaps and opportunities in these areas. Section 3 reviews related work in the fields of ontology integration, prompt tuning, and abstractive summarization. Section 4 presents the proposed methodology for ontology-based prompt tuning in news article summarization. Section 5 details the experimental setup and results, followed by a discussion of the results in Section 6. Finally, Section 7 concludes the paper and suggests directions for future research.

## 2 Background

Ontology, a formal representation of knowledge within a specific domain, plays a crucial role in enhancing various NLP tasks, including information retrieval, semantic search, and knowledge extraction (Rudnicki et al., 2018). Ontologies capture domain-specific knowledge, including concepts, relationships, and constraints, providing structured frameworks that facilitate knowledge sharing and semantic interoperability. In text summarization, ontology integration can offer significant benefits by guiding and improving the summarization process. However, challenges such as knowledge representation, alignment with textual data, and the dynamic nature of news content make ontology integration in summarization models complex (Wiebe and Chan, 2012). While ontologies have proven valuable in data management and decision-making, their application in summarization remains underexplored (Grimm et al., 2011; Elsayed, 2023).

Prompt generation and tuning are essential for optimizing summarization model performance. Crafting effective prompts requires a deep understanding of the target domain and input text characteristics, ensuring that the prompts are concise and contextually relevant. Prompt tuning, involving iterative refinement, adjusts prompts based on evaluation metrics and feedback to enhance summary quality (Zhang et al., 2017). However, challenges arise in crafting prompts that capture text complexity while remaining concise and in tuning them to avoid overfitting, ensuring generalization across different datasets (Ma et al., 2023). Overcoming these challenges is vital for advancing text summarization capabilities.

Text summarization, a critical task in NLP (Dong et al., 2019), involves condensing large volumes of text into concise, informative summaries (Mieskes, 2022). Traditional methods are categorized into extractive and abstractive techniques (Habu et al., 2023). Extractive summarization selects significant portions of the source text, preserving original phrasing but often struggling with coherence (Moratanch and Chitrakala, 2017; Rahimi et al., 2017; Yadav et al., 2023). Abstractive summarization generates novel sentences that convey the text’s essence, producing more coherent and contextually relevant summaries (Yeasmin et al., 2017). However, abstractive summarization faces challenges in maintaining factual accuracy and fluency, necessitating sophisticated model architectures and fine-tuning strategies (Kumar and Solanki, 2023; Rush et al., 2015a).

## 3 Related work

This section provides a comprehensive overview of significant contributions to the field, focusing on extractive and abstractive summarization approaches, as well as the integration of ontology and prompt tuning techniques. A summary of the literature review can be seen on Table 1.

**Table 1:** Summary of literature review including their proposed work, findings, and limitations.

Reference #	Proposed work	Findings	Limitations
Srikanth et al. (2020)	An automatic extractive text summarization system for biomedical review papers using a graph-based method, PageRank.	Enhanced with UMLS ontology and WMD word embedding to establish sentence connections.	The study remained confined to extractive methods and did not explore potential improvements through advanced language models.
Madhuri and Kumar (2019)	A statistical extractive summarization method that ranked sentences based on frequency and converted summaries to audio.	Demonstrated improved accuracy over conventional methods.	Did not explore integration of large language models or advanced techniques, focusing instead on frequency-based sentence selection.
Majeed and Kala (2023)	A model for extractive summarization using sentence ranking and the TextRank algorithm.	Generated grammatically correct summaries by evaluating phrase importance and compiling significant sentences.	Lacked smooth readability due to the algorithm’s emphasis on sentence importance without integrating semantic coherence.
Srikanth et al. (2020)	BERT for extractive summarization by clustering sentence embeddings with K-means, incorporating reference resolution.	Introduced a dynamic method for selecting the optimal number of sentences to enhance summary quality.	Challenges in ensuring coherence and contextual relevance across diverse educational content.
Moawad and Aref (2012)	An abstractive summarization method through a semantic graph reduction technique.	Produced paraphrased and contextually relevant summaries.	Limited by its use of a fixed dataset and document size, impacting generalizability.
Harivignesh et al. (2022)	Abstractive summarization using BERT and a pointer-generator network under the T5 framework.	Showed superior performance, surpassing existing summarizers by up to 6 ROUGE points.	Faced data loss from exclusion of sentences without geographic information, needing integration of graph-based algorithms.
Nallapati et al. (2016)	The switching generator/pointer model on the Gigaword corpus.	The model accurately employed pointers for named entities and multi-word phrases.	Overall performance improvement was marginal, with a potential for more significant impact in scenarios with a heavier tail distribution.
Patel et al. (2020)	A model for generating concise summaries from SERPs using Seq2Seq with LSTM networks for encoding and decoding.	Demonstrated promising results in generating meaningful summaries from search engine results.	Faces limitations in handling highly diverse or noisy data from multiple sources, affecting coherence and relevance.
Rush et al. (2015b)	A neural attention-based model for abstractive summarization, integrating a probabilistic framework with a generation algorithm.	Produced accurate and contextually relevant summaries.	Challenges in achieving consistently high grammatical quality and handling longer texts.
Radford et al. (2019)	Prompt-tuned article summarization using large language models like GPT-3.	Generated concise, coherent, and contextually relevant summaries through iterative prompt tuning.	Challenges in maintaining grammatical consistency and handling complex text structures.
Zhang and Liu (2022)	Intent-aware Prompt Learning (InPL) for medical question summarization.	Achieved notable performance improvements, establishing new state-of-the-art results.	Absence of specialized medical knowledge in pre-training language models, which could enhance summarization for medical content.
Liu and Lapata (2019), Ziegler et al. (2020), Keskar et al. (2019), Gao et al. (2021)	Few-shot learning, conditional transformers, and human preference-based fine-tuning for summarization.	Showcased the effectiveness of pre-trained encoders and prompt-tuning techniques for high-quality summarization.	Ongoing need for iterative improvements to refine summarization outputs based on specific criteria and minimal data adjustments.
Song et al. (2017), Li et al. (2019)	Ontology-driven summarization aligning with underlying knowledge structures.	Enhanced readability and informativeness of summaries through ontology alignment.	Integration of domain-specific knowledge while maintaining contextual relevance across diverse domains remains challenging.

### 3.1 Extractive summarization

Extractive summarization, which involves selecting and compiling key sentences from the source text, has been extensively studied and refined. A previous study investigated an automatic extractive text summarization system specifically for biomedical review papers. Their system utilized a graph-based method, PageRank, enhanced with UMLS ontology and WMD word embedding to establish sentence connections. Despite these advancements, the study remained confined to extractive methods, not exploring potential improvements through advanced language models (Yongkiatpanich and Wichadakul, 2019).

Another paper proposed a statistical extractive summarization method that ranked sentences based on frequency and selected the top-ranked sentences for inclusion in the summary. This model, which also converted summarized text into audio format, demonstrated improved accuracy over conventional methods. However, the research did not explore the integration of large language models or advanced techniques, focusing instead on frequency-based sentence selection (Madhuri and Kumar, 2019).

Another study focused on developing a model for extractive text summarization using sentence ranking and the TextRank algorithm. The model demonstrated effective performance in generating extractive summaries by evaluating the importance of phrases and compiling the most significant sentences. However, while the TextRank algorithm produced grammatically correct summaries, the sequence of sentences often lacked smooth readability. This limitation arose from the algorithm’s emphasis on sentence importance without integrating semantic coherence, suggesting a need for further refinement to enhance the fluidity and contextual relevance of the summaries (Majeed and Kala, 2023).

The paper leveraged the BERT model to perform extractive summarization by clustering sentence embeddings with K-means and introduced a dynamic method for selecting the optimal number of sentences. They also incorporated reference resolution and adjustments to summary length based on content to enhance summary quality. However, challenges remain in ensuring the coherence and contextual relevance of summaries across diverse educational content (Srikanth et al., 2020).

### 3.2 Abstractive summarization

Abstractive summarization techniques generate summaries by paraphrasing and producing new sentences, aiming for greater readability and contextual relevance (Chen and Bansal, 2018; Liu et al., 2022; Paulus et al., 2017). A previous study introduced an innovative method for generating abstractive summaries through a semantic graph reduction technique was limited by its use of a fixed dataset and document size, which may impact the generalizability of the findings (Moawad and Aref, 2012).

Another paper explored abstractive summarization using the BERT model and a pointer-generator network, following the Text-to-Text Transfer Transformer (T5) framework. Their system showed superior performance, surpassing existing summarizers by up to 6 ROUGE points on the CNN/Daily Mail dataset (Harivignesh et al., 2022). While this approach significantly enhanced summarization quality and information retrieval speed, it encountered limitations related to data loss from the exclusion of sentences without geographic information. This highlighted the need for integrating graph-based algorithms to maintain comprehensive text relationships (Harivignesh et al., 2022; See et al., 2017).

Nallapati et al. (2016), investigated the application of the switching generator/pointer model on the Gigaword corpus, revealing that the model accurately employed pointers for both named entities and multi-word phrases. However, despite this precision, the overall performance improvement was marginal. However, the model’s impact could be more significant in scenarios with a heavier tail distribution of rare words.

Patel introduces a model utilizing abstractive summarization techniques to generate concise summaries from search engine results pages (SERPs). Their approach leverages the Seq2Seq architecture with Long Short-Term Memory (LSTM) networks for encoding and decoding to produce coherent and contextually accurate summaries. The approach involves extracting data from various sites, processing it through the Seq2Seq model, and presenting a synthesized summary to the user. While the model demonstrates promising results in generating meaningful summaries, it faces limitations in handling highly diverse or noisy data from multiple sources, which may affect summary coherence and relevance (Patel et al., 2020).

Another study developed a neural attention-based model for abstractive summarization, leveraging recent advances in neural machine translation by integrating a probabilistic framework with a generation algorithm, resulting in accurate and contextually relevant abstractive summaries. Despite these advancements, the current model faces challenges in achieving consistently high grammatical quality and effectively handling longer texts (Rush et al., 2015b).

### 3.3 Prompt generation and tuning

Prompt-tuned article summarization represents an advanced technique in natural language processing (NLP) that leverages prompt guidance to enhance the quality of generated summaries (Allahyari et al., 2017; Clariso and Cabot, 2023; Lester et al., 2021). This method involves refining and tuning prompts to generate high-quality summaries, particularly effective for dynamic and diverse news content (Brown et al., 2020; Ma et al., 2022). By iteratively adjusting prompts, this approach harnesses the capabilities of pre-trained language models such as GPT-3, delivering concise, coherent, and contextually relevant summaries (Radford et al., 2019).

Zhang and Liu presented a novel Intent-aware Prompt Learning (InPL) method tailored for medical question summarization achieving notable performance improvements on two medical question summarization datasets, establishing new state-of-the-art results (Zhang and Liu, 2022). However, a key limitation was the absence of incorporating specialized medical knowledge into the pre-training language models, which could further enhance the summarization process for medical content.

Brown et al. (2020) demonstrated the capabilities of GPT-3 in few-shot learning, highlighting the potential of large language models for prompt tuning in summarization tasks. Radford et al. discussed the unsupervised multitask learning capabilities of language models, underscoring their adaptability in diverse summarization scenarios (Radford et al., 2019). Another research showcased the effectiveness of pre-trained encoders in text summarization, illustrating the benefits of leveraging existing knowledge for generating high-quality summaries (Liu and Lapata, 2019). Ziegler et al. (2020) emphasized the importance of fine-tuning language models based on human preferences, noting the significance of iterative improvements in summary quality. Keskar et al. (2019) introduced conditional transformers for controllable generation, offering techniques for refining summarization outputs based on specific criteria. Gao et al. (2021) focused on enhancing few-shot learning in pre-trained models, further advancing prompt-tuned summarization through minimal data adjustments.

### 3.4 Ontology integration

Ontology-driven summarization offers distinct advantages over traditional summarization methods (Li et al., 2018; Wang et al., 2018, 2019; Hennig et al., 2008), which often rely on statistical or heuristic-based techniques to identify key sentences or phrases (Zhang et al., 2022). While effective to some degree, these conventional methods may overlook deeper semantic relationships and contextual nuances within the text (Bazrafshan et al., 2019).

In contrast, ontology-driven prompt generation represents a sophisticated approach to text summarization that leverages domain-specific knowledge representations to guide and enhance the summarization process. By mapping news articles to an ontology, researchers and practitioners can identify and analyze relevant entities, relationships, and concepts within the text, thereby generating targeted prompts that effectively capture the key information and salient points of the article (Gomez-Verdejo et al., 2016; Ragunath and Sivaranjani, 2015).

By aligning the summarization process with the underlying knowledge structure provided by the ontology, summaries become more coherent and consistent, thereby enhancing their readability and informativeness (Song et al., 2017; Li et al., 2019).

Despite the significant advancements in ontology-driven summarization and prompt generation, several limitations persist that warrant ongoing research and development. One of the primary challenges is maintaining grammatical consistency in the generated summaries, especially when dealing with diverse and complex text structures. This issue is compounded by the difficulty of integrating domain-specific knowledge in a manner that remains both accurate and contextually relevant across varying domains (Schneider and Šimkus, 2020). Furthermore, the dynamic nature of news content poses additional challenges, as ontologies may struggle to keep pace with emerging terminology and concepts. Addressing these limitations is essential for further refining summarization methodologies, ultimately enhancing their effectiveness, applicability, and ability to produce coherent and informative summaries across a broad range of contexts.

## 4 Methodology

**Figure 1:**
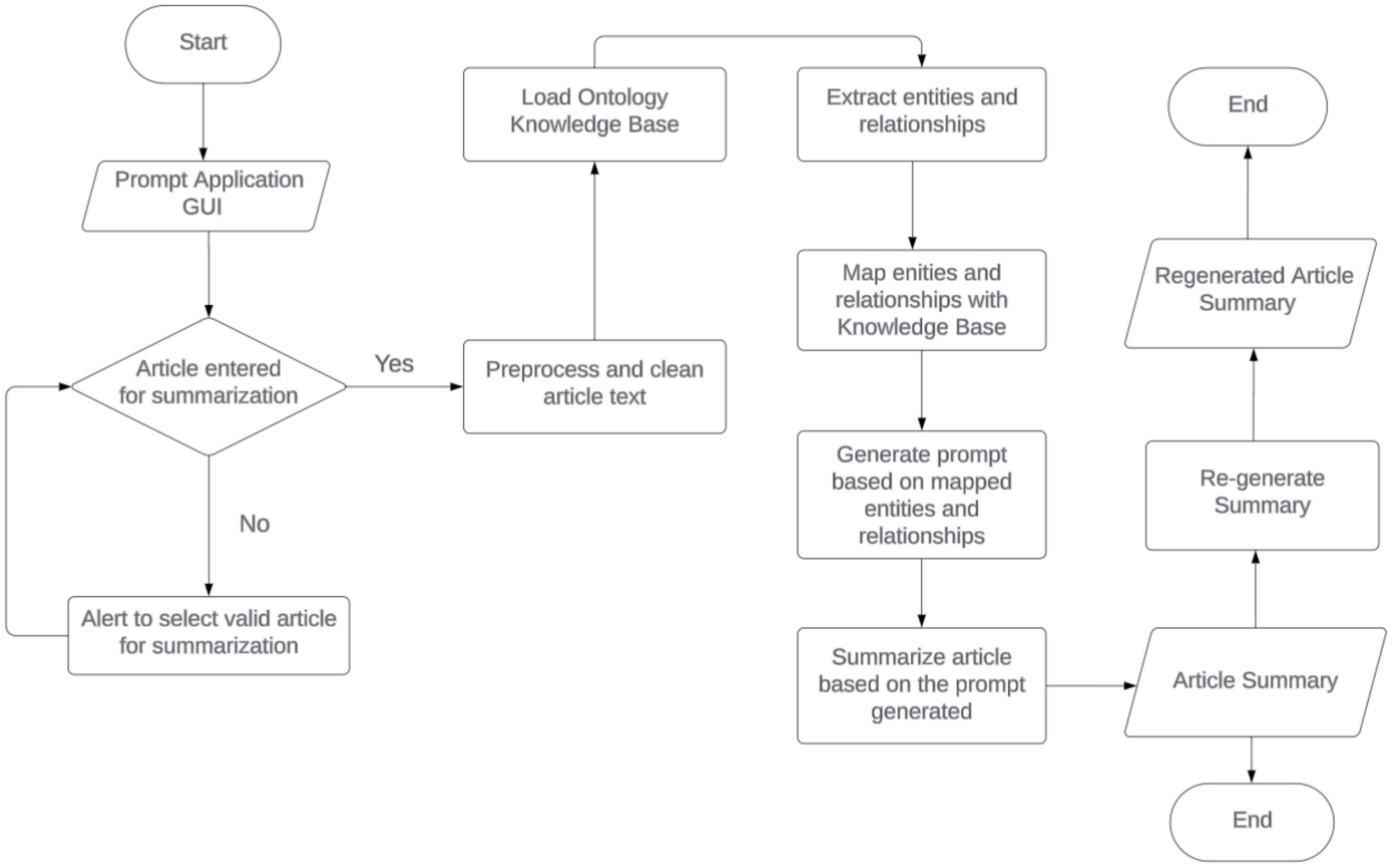
System process flow.

This section explains the systematic approach employed to develop and evaluate an ontology-based prompt tuning framework for news article summarization, integrating advanced NLP techniques with domain-specific knowledge to enhance summary quality and relevance as shown by Figure 1.

### 4.1 Data collection and preprocessing

For this study, selecting datasets that encompass a diverse range of news topics, rich linguistic features, and structured metadata is essential for ontology-based tuning and ensuring the accuracy and coherence of the generated summaries (Gupta and Lehal, 2010; Mahmood, 2017; Misra, 2022; Xu et al., 2023).

In this research, the CNN/DailyMail Dataset (2015) was chosen for its extensive collection of articles and summaries, which provides a robust foundation for training and fine-tuning the summarization model across various domains. This dataset includes approximately 300,000 tuples, offering a comprehensive resource for both supervised learning and evaluation (Dong et al., 2018). The inclusion of annotations for summarization facilitates the supervised learning process, allowing the model to adapt to the specific nuances of different topics (Nallapati et al., 2016).

In this study, the preprocessing of articles and summaries is meticulously aligned with the requirements of the T5 model. A dedicated function is employed to prepend the prompt “summarize:” to each article, ensuring that the T5 model recognizes the task. Both the input and target (summary) texts are tokenized using the T5 tokenizer. The input sequences are truncated to 800 tokens, reflecting the mean token count of 781 for the articles, while the target sequences are truncated to 150 tokens. This preprocessing step is crucial for optimizing the dataset, which is subsequently divided into training and validation sets for effective model training and evaluation.

### 4.2 Ontology integration and analysis

In this study, ontological analysis plays a pivotal role in enhancing the effectiveness of summarization by ensuring the accurate identification and understanding of key entities and their interactions within the text.

**Figure 2:**
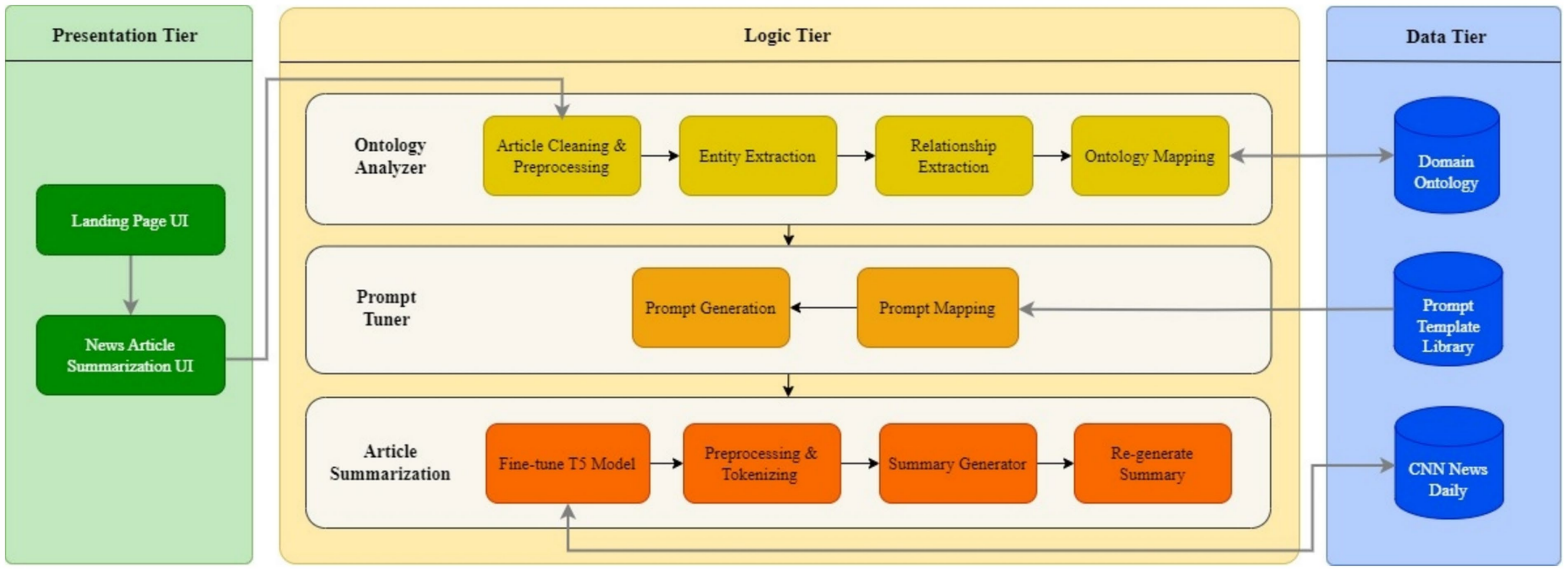
High-level architecture.

**Figure 3:**
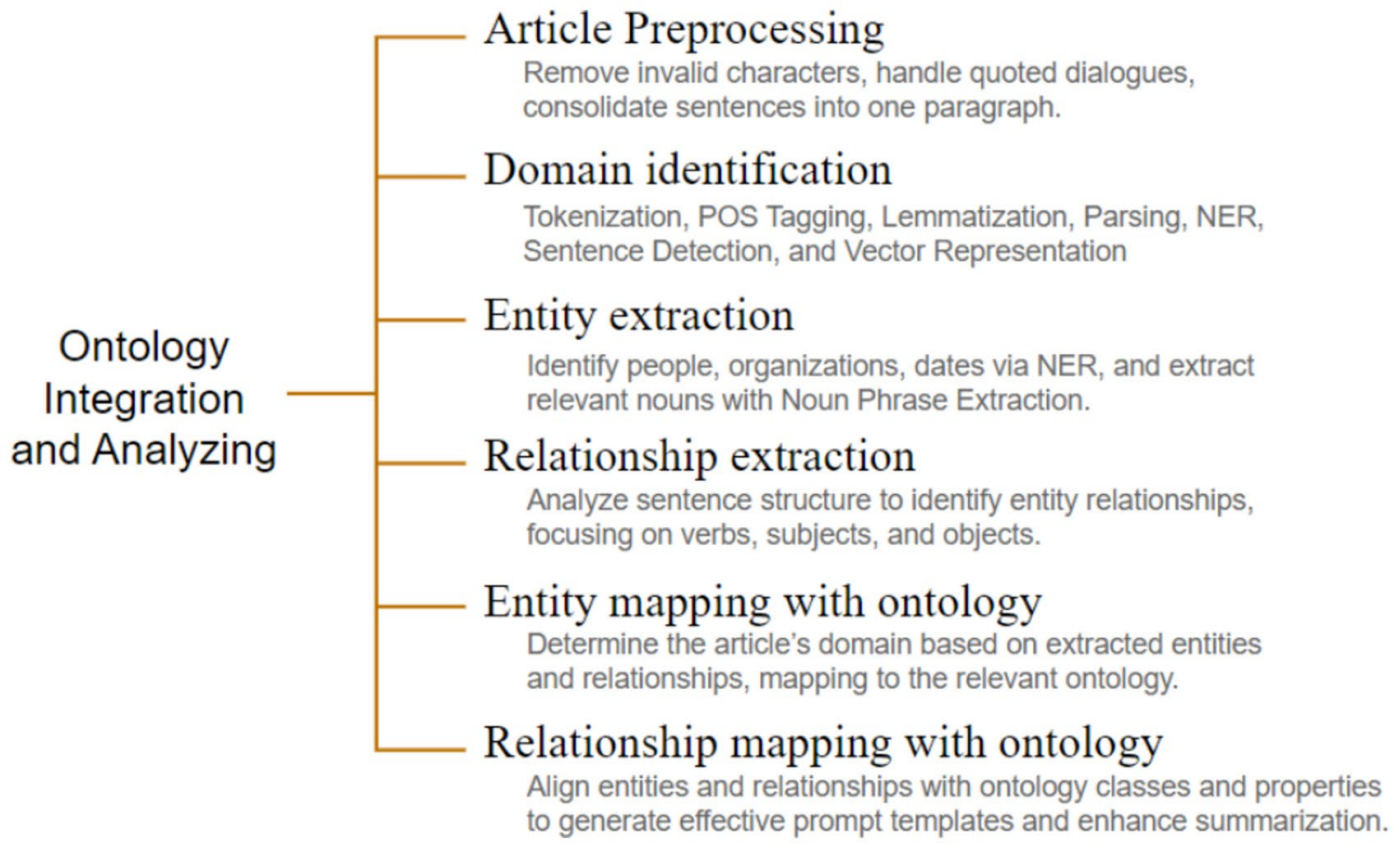
Ontology integration and analyzing workflow.

The ontology is integrated and used in analyzing the article text as a series of steps as shown in Figure 2. The process begins with Article Cleaning, a crucial step in preparing the raw article text for further processing. During this phase, the text is meticulously refined to remove any invalid characters, such as special symbols, extraneous whitespace, and HTML tags that may have been carried. Additionally, quoted dialogues and attributions from key figures are handled carefully to maintain clarity and ensure they are correctly formatted. This step helps ensure that the text is in a clean, structured format, free from distractions and inconsistencies, which is essential for the subsequent stages of natural language processing, such as tokenization, entity recognition, and summarization. By addressing these details, the cleaning process ensures the text is ready for accurate and efficient analysis. Article Preprocessing employs comprehensive natural language processing techniques, including Tokenization, Part-of-Speech Tagging, Lemmatization, Dependency Parsing, Named Entity Recognition (NER), Sentence Boundary Detection, and Vector Representation, which are essential for extracting linguistic features necessary for effective summarization. Entity extraction plays a critical role in identifying and categorizing key elements within the text, enabling the system to construct a foundational understanding of the content. This step employs Named Entity Recognition (NER) techniques to accurately identify entities such as people, organizations, locations, dates, and other domain-specific terms that are central to the article. Beyond NER, Noun Phrase Extraction is employed to identify additional relevant nouns and noun phrases that may not fall under standard entity categories but are pivotal for contextual understanding. This dual approach ensures a thorough identification of both explicit and implicit key content. Relationship extraction focuses on understanding how the identified entities interact within the text. This step analyzes sentence structures using advanced syntactic and semantic parsing techniques. By examining verbs and their associated subjects and objects, the system identifies relationships such as actions, associations, and dependencies between entities. Subsequently, Domain Extraction categorizes the article into a specific thematic domain by analyzing the extracted entities and relationships. This step leverages ontological mappings to evaluate the significance and context of the identified elements. This Entities and Relationships Mapping process aligns the identified entities and relationships with ontology classes and properties, facilitating the creation of precise prompt templates and categorization, thereby enhancing the summarization accuracy. Figure 3 denotes the steps involved in the Ontology integration module along with the sub-steps being executed.

Utilizing ontologies facilitates the structuring of information by defining the relationships between various concepts within a domain (Chen et al., 2020). Key criteria for constructing an accurate ontology include comprehensive coverage of domain-specific concepts, clear hierarchical relationships, and well-defined properties and attributes (Noy and McGuinness, 2001). When mapping the news article to the ontology, it is vital to consider the relevance of the article’s content to the ontology’s concepts, the precision of concept matching, and the effective handling of ambiguities and synonyms. Developing a robust ontology is essential for ensuring that the summarization model can accurately interpret and summarize the content, leading to more precise and meaningful summaries (Staab et al., 2009). An inadequate ontology could lead to misinterpretation of the article’s context, thereby diminishing the overall quality and applicability of the summarization in real-world scenarios (Buitelaar et al., 2005).

### 4.3 Prompt tuning

Prompt tuning is integral to this research, facilitating the development of precise and contextually relevant prompts that direct the summarization model. By mapping entities and relationships derived from the ontology analysis to domain-specific prompt templates, the model generates concise and relevant summaries. This methodology supports iterative improvement, refining the accuracy and contextual appropriateness of summaries over time (Liu et al., 2022).

**Figure 4:**
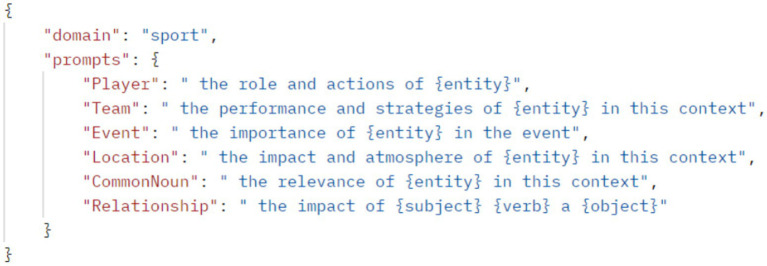
Sample prompt template.

In this study, prompt tuning involves several key steps to optimize the summarization process. Prompt Template Identification utilizes a function to access and filter domain-specific prompt templates from a JSON file, based on the domain information derived from ontology analysis. This approach ensures the model uses relevant domain-specific language patterns, enhancing the relevance and accuracy of summaries. Entity Prompt Mapping is a critical step in structuring the extracted information into a format that can effectively guide the summarization model. This step begins with categorizing entities into predefined labels such as ‘Person’, ‘Organization’, ‘Date’, ‘Location’ and other domain-specific categories relevant to the article. Once categorized, these entities are transformed into prompts using predefined templates designed to encapsulate their context and details accurately (as shown by Figure 4). Templates are carefully crafted to maintain the semantic integrity of the information, ensuring that the entities are represented in a manner that reflects their role and significance in the article. This structured formatting not only organizes the data but also prepares it for seamless integration into the summarization pipeline.

Relationship Prompt Mapping focuses on representing the interactions between entities in a structured format. This step begins by analyzing the identified relationships, which are typically defined by verbs and their associated subjects and objects. These relationships are then translated into prompts using predefined templates tailored to the specific nature of the interaction. A relationship prompt for the example sentence might be formatted as shown in Figure 4. The use of templates ensures consistency in how relationships are represented, providing a clear and coherent structure for the summarization model to follow.

Finally, Summarization Prompt Generation integrates the entity and relationship prompts into a cohesive final prompt that serves as the input for the summarization model. This step involves combining the individual prompts into a narrative structure that aligns with the article’s overall context and domain. By combining entity and relationship prompts, this step ensures that the final input to the summarization model is both comprehensive and contextually rich. This structured approach not only enhances the quality of the generated summaries but also aligns them with the objectives of ontology-based prompt tuning.

### 4.4 Summary generation

In this study, summary generation is implemented utilizing T5 Hugging Face Transformers, which is central to generating concise and coherent summaries that effectively capture the essence of news articles. Unlike extractive summarization, which relies on selecting and rephrasing existing sentences, abstractive techniques leverage natural language understanding to produce summaries through paraphrasing and creative rephrasing of the input text (Lewis et al., 2019). Hugging Face Transformers provides a robust suite of pre-trained models and fine-tuning capabilities, which are instrumental in developing task-specific abstractive summarization systems (Wolf et al., 2020; Harivignesh et al., 2022). Notably, the T5 model, introduced by Raffel et al. (2023), exemplifies exceptional performance in various natural language processing tasks, including summarization.

As shown in Algorithm 1, optimizing the T5 model’s performance requires configuring training parameters through TrainingArguments(). This setup includes specifying critical parameters such as learning rate, batch size, number of epochs, and evaluation strategy. The training is orchestrated by the Trainer class, which manages the training loop, evaluation, and logging. The hyperparameters for model training were meticulously configured to optimize performance and efficiency.

The evaluation_strategy was set to steps, enabling evaluation every 2,000 steps to monitor progress while minimizing interruptions. The learning rate was fixed at 3e-4, promoting gradual convergence. Both per_device_train_batch_size and per_device_eval_batch_size were set to 16 to expedite training processes. Gradient accumulation was utilized with gradient_accumulation_steps set to 2 to emulate a larger batch size and stabilize training. The use of fp16 enabled 16-bit precision, enhancing computational speed and reducing memory usage. Finally, the best model, based on evaluation loss, was loaded at the end of training through load_best_model_at_end and ensure the model’s optimal performance using metric_for_best_model.

#### ALGORITHM 1 Fine-tune summarization model


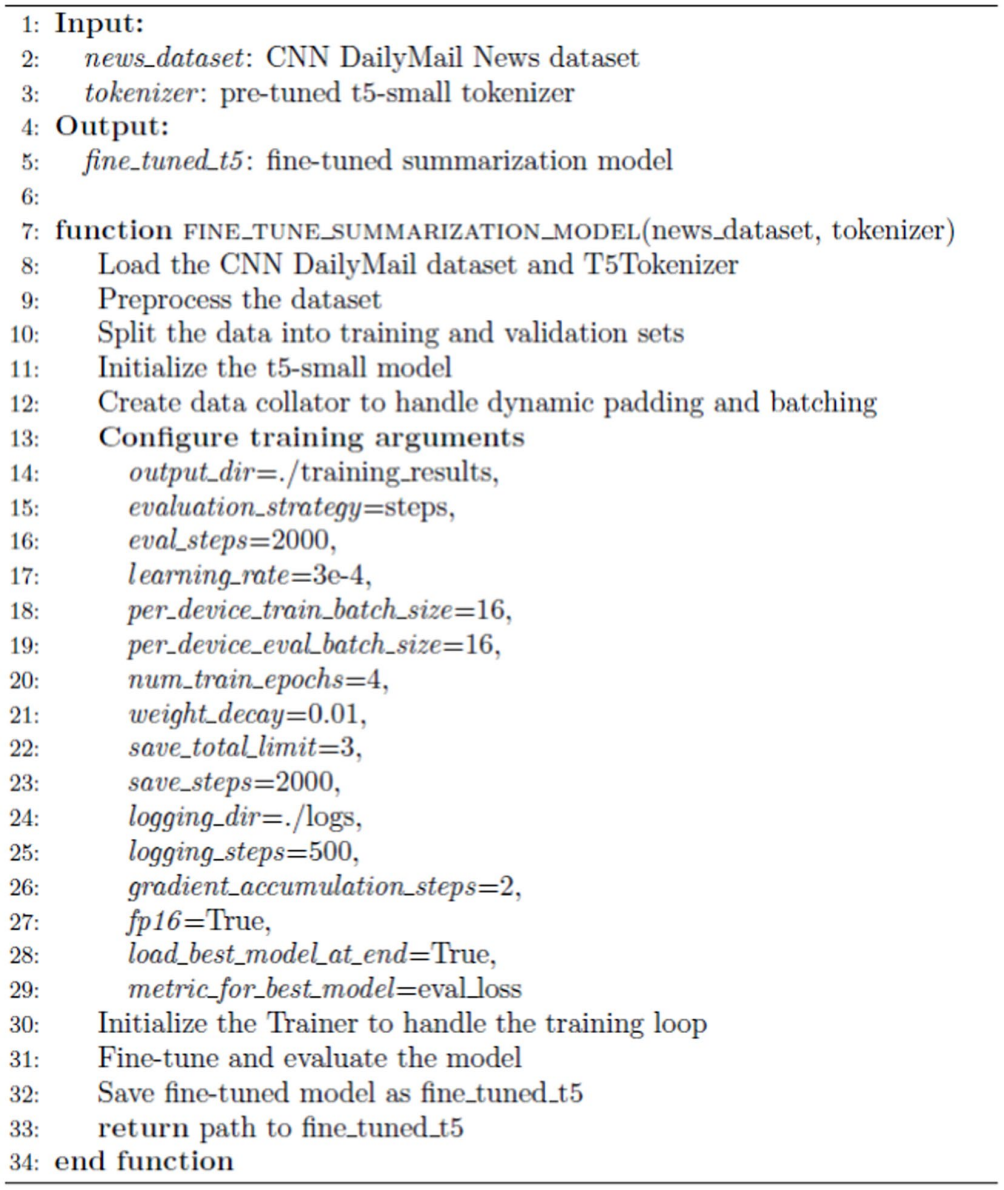


The model’s performance is assessed using model_trainer.evaluate(), which involves a forward pass through the evaluation dataset to generate predictions and calculate performance metrics, including loss and task-specific metrics such as ROUGE scores. This evaluation step is crucial for determining the model’s generalizability to new data and guiding further training or fine-tuning.

#### ALGORITHM 2 Summarize news article


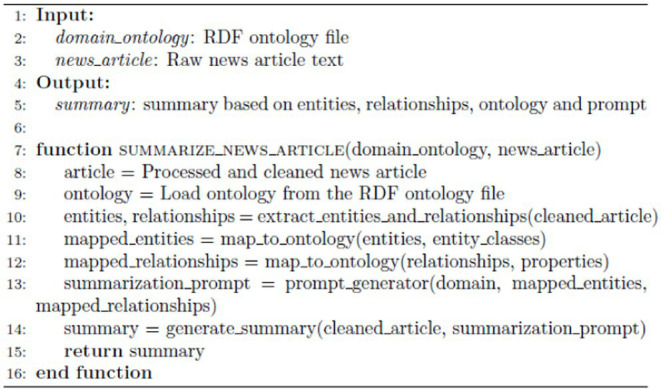


The innovation shown by the Algorithm 2 for summarization is its integration of domain ontology with summarization through prompt tuning. Utilizing an RDF ontology file, the algorithm enhances the summarization process in several keyways. First, it embeds domain-specific context by incorporating knowledge from the RDF ontology, thereby refining the extraction and mapping of entities and relationships to ensure that the summaries adhere to domain-specific terminologies and relationships.

Second, the structured mapping of entities and relationships to ontology classes and properties introduces an additional layer of structure and relevance, enhancing the accuracy and coherence of the generated summaries. Lastly, the algorithm employs a dynamically generated prompt, derived from the ontology and mapped entities, to direct the summarization model’s focus, resulting in summaries that are more contextually precise and informative. This approach uniquely integrates entity extraction, ontology mapping, and prompt-based summarization to generate summaries that are both domain-relevant and well-structured.

## 5 Experiments and results

The experiments aim to rigorously assess the summarization model’s ability to generate high-quality summaries was systematically evaluated by benchmarking its outputs against industry standards and real-world data. The adaptability of the system to varying article lengths and evolving content was tested, confirming its ongoing relevance and operational efficacy. The integration of the ontology-based prompt tuning approach was also scrutinized to verify its seamless and intended operation within the overall architecture.

### 5.1 Experimental settings

The model was fine-tuned over num_train_epochs of 4 epochs to ensure effective learning. A weight decay of 0.01 was applied to mitigate overfitting. Checkpoints were managed with save_total_limit set to 3 and save_steps set to 2000 to balance progress tracking with disk space conservation.

Summarization is achieved by applying a domain-specific prompt and tokenizing the input with a maximum length of 1,024 tokens. The T5 model generates the summary using beam search with num_beams set to 6, while enforcing length constraints (min_length = 30, max_length = 150) and penalties to prevent repetition (no_repeat_ngram_size = 2, repetition_penalty = 1.2). The summary IDs are decoded to produce a coherent and contextually accurate summary.

### 5.2 Metrics

In evaluating the performance of our summarization model, we utilized a combination of ROUGE, BERTScore, and BARTScore metrics to provide a comprehensive assessment of summarization quality (Stewart et al., 2003). While BLEU and METEOR are commonly used in machine translation, they primarily rely on exact word matching, which may not capture the semantic richness of abstractive summaries hence have been omitted in this evaluation.

- *ROUGE:* ROUGE-1, ROUGE-2, and ROUGE-L were selected for their ability to capture various dimensions of summarization effectiveness, as demonstrated in prior studies (Rajalakshmy and Remya, 2016). These metrics are instrumental in measuring n-gram overlap and the overall linguistic quality of the summaries (Lin, 2004).
- *BERTScore:* We employed BERTScore to evaluate the model’s accuracy, completeness, and balance in generating summaries that effectively capture and convey relevant information (Kulkarni et al., 2021).
- *BARTScore*: Was utilized to assess the fluency, relevance, coherence, and faithfulness of the generated text, offering a nuanced evaluation framework that addresses limitations inherent in traditional metrics like BLEU (Papineni et al., 2002) and ROUGE (Yuan et al., 2021).

These combined metrics provide a robust foundation for evaluating the summarization model’s performance across multiple dimensions of quality.

### 5.3 Baselines

In our research, we selected BART, BERT, GPT 3.5 Instruct, and GPT 3.5 Turbo as baseline models for comparison due to their prominence in the field of natural language processing (NLP) and summarization by carefully considered each model’s architecture, strengths, and typical use cases to ensure a well-rounded evaluation of our ontology-based prompt tuning approach. BART and BERT are widely recognized for their robust language understanding and generative capabilities, making them ideal for benchmarking against our model’s performance in abstractive summarization. GPT 3.5 Instruct and GPT 3.5 Turbo were included to provide insights into how our model fares against the latest advancements in large language models, particularly those optimized for instruction-based tasks. These baselines were chosen to cover a broad spectrum of methodologies, from sequence-to-sequence models like BART to transformer-based architectures like BERT and GPT-3.5, ensuring a comprehensive evaluation.

The comparison criteria centered on key performance metrics such as Precision, Recall, F1 Score, ROUGE, BERTScore, and BARTScore, which offer a multidimensional assessment of summarization quality. However, using these baseline models posed challenges, particularly in aligning them with our domain-specific prompt tuning approach. Each model has distinct strengths, but they also exhibit limitations, such as BERT’s reduced efficacy in generative tasks and GPT-3.5’s computational demands. Despite these challenges, their inclusion is justified as they represent the current state-of-the-art, providing a relevant benchmark for evaluating the improvements introduced by our ontology-based prompt tuning method. This selection allows for a rigorous comparison, highlighting the advancements and potential areas for improvement in our approach.

### 5.4 Results

The results of our experiments provide a detailed analysis of the summarization model’s performance, highlighting its strengths and areas for improvement across various metrics. By systematically comparing our model to existing state-of-the-art (SOTA) techniques for summarization, we demonstrate the efficacy in generating high-quality summaries with this study.

**Figure 5:**
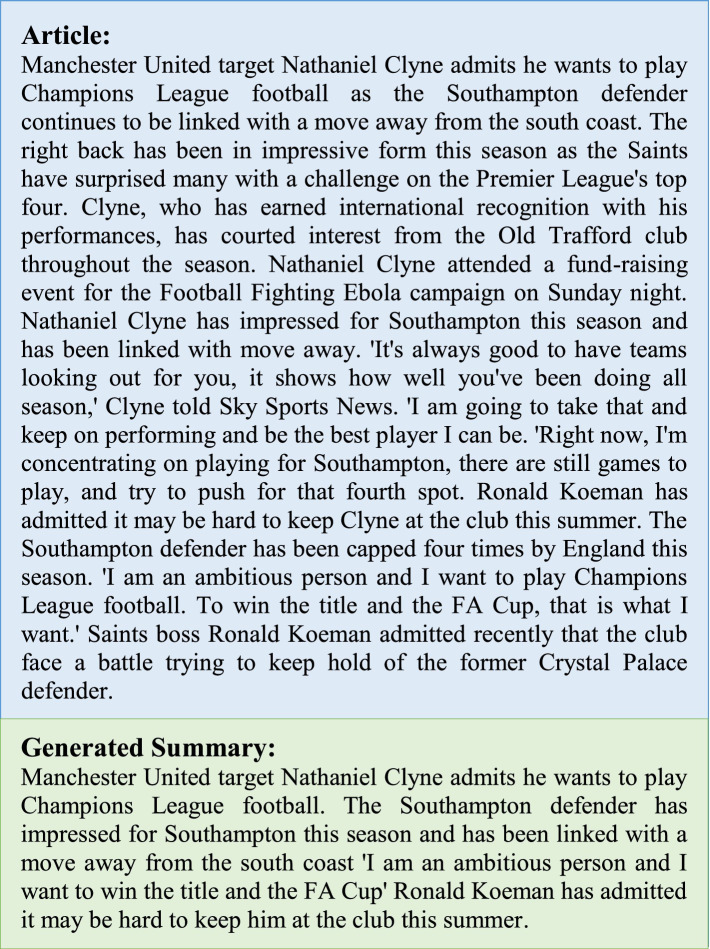
Sample article and summary generated.

Figure 5 shows an article taken from the CNN/Daily Mail evaluation split along with the summary generated using the ontology-based prompt tuning approach for summarization.

Table 2 holds the results our model obtained against the reference summary along with the results of other SOTA models.

**Table 2:** Model comparison of F1, ROUGE and BART scores.

	BART	BERT	GPT 3.5 instruct	GPT 3.5 turbo	Our Model
Precision	0.7018	0.7147	**0.8113**	0.7690	0.8090
Recall	**0.7513**	0.6499	0.6581	0.7378	0.7235
F1	0.7257	0.6808	0.7267	0.7531	**0.7639**
ROUGE-1	0.5531	0.4403	0.4227	0.5207	**0.5828**
ROUGE-2	0.2473	0.2165	0.2923	0.2874	**0.3893**
ROUGE-L	0.3191	0.3144	0.3030	0.375	**0.4370**
BARTScore	0.0489	0.0494	0.0491	**0.0509**	0.0495

The highlighted values in this table represent the highest score achieved for each evaluation metric.

#### 5.4.1 F1 score

(1) $$F1=2\cdot \frac{P\cdot R}{P+R}$$

The evaluation of summarization models, including BART, BERT, GPT 3.5 instruct, GPT 3.5 turbo and our proposed model, reveals significant insights into their performance across Precision, Recall, and F1 metrics. The results indicate that our model achieved a Precision score of 0.8090, a Recall score of 0.7235, and an F1 score of 0.7639, outperforming several baseline models. Specifically, BERT, while having a competitive Precision score of 0.7147, showed lower Recall (0.6499) and F1 (0.6808) compared to our model. GPT 3.5 instruct and GPT 3.5 turbo also demonstrated notable performance, with F1 scores of 0.7267 and 0.7531, respectively. However, our model’s superior balance of Precision and Recall highlights its effectiveness in generating high-quality summaries. These results underscore the robustness of our approach in capturing and presenting relevant information, and the potential advantages of our model over existing techniques in the context of abstractive summarization.

#### 5.4.2 ROUGE scores

(2) $$R_{LCS}=\frac{LCS\left(X,Y\right)}{m}$$

(3) $$P_{LCS}=\frac{LCS\left(X,Y\right)}{n}$$

(4) $$F_{LCS}=\frac{\left(1+\beta^{2}\right)\cdot P_{LCS}\cdot R_{LCS}}{R_{LCS}+\beta^{2}\cdot P_{LCS}}$$

The evaluation of our model using ROUGE metrics demonstrates its effectiveness in text summarization tasks. Specifically, our model achieved a ROUGE-1 score of 0.58, reflecting a high level of unigram overlap with reference summaries. This indicates that our model effectively captures key terms and maintains sequence coherence, surpassing BERT and GPT-3.5, and performing comparably to BART. In terms of ROUGE-2, which measures bigram overlap, our model recorded a score of 0.38, outperforming BART, BERT, and GPT-3.5 Instruct, highlighting its proficiency in preserving pairs of consecutive words. The ROUGE-L score of 0.44 underscores the model’s ability to capture the overall structure and ordering of words, demonstrating enhanced fluency and coherence relative to other state-of-the-art (SOTA) models. Collectively, the results show that our model excels in unigram overlap and sequence preservation, outperforming several SOTA models, including BART and BERT, in retaining essential content and maintaining coherence. However, there remains potential for improvement in bigram overlap. Overall, these findings suggest that our model provides accurate and effective summarization, closely aligning with reference summaries in both content and sequence.

#### 5.4.3 BART scores

(5) $$\mathrm{BARTScore}=\sum_{t=1}^{m}\omega_{t}\log p\left(y_{t}|y_{<t},x,\theta \right)$$

The evaluation of ontology-based prompt tuning through BARTScore reveals a competitive performance compared to leading summarization techniques. Our model achieved a BARTScore of 0.0495, which is slightly higher than BERT and on par with GPT-3.5 Instruct. Although it does not surpass GPT-3.5 Turbo, it demonstrates a strong capability in generating summaries that align closely with reference texts. This performance indicates that our model is effective in comparison to other state-of-the-art (SOTA) methods. The BARTScore measures summary quality in terms of fluency and coherence, and our model’s results suggest that the integration of ontology-based prompt tuning positively influences these aspects. This supports the effectiveness of our approach in improving summary fluency and coherence. While our model aligns well with reference summaries, there is still potential for enhancement to match or exceed the performance of the leading models.

#### 5.4.4 Valuation

**Figure 6:**
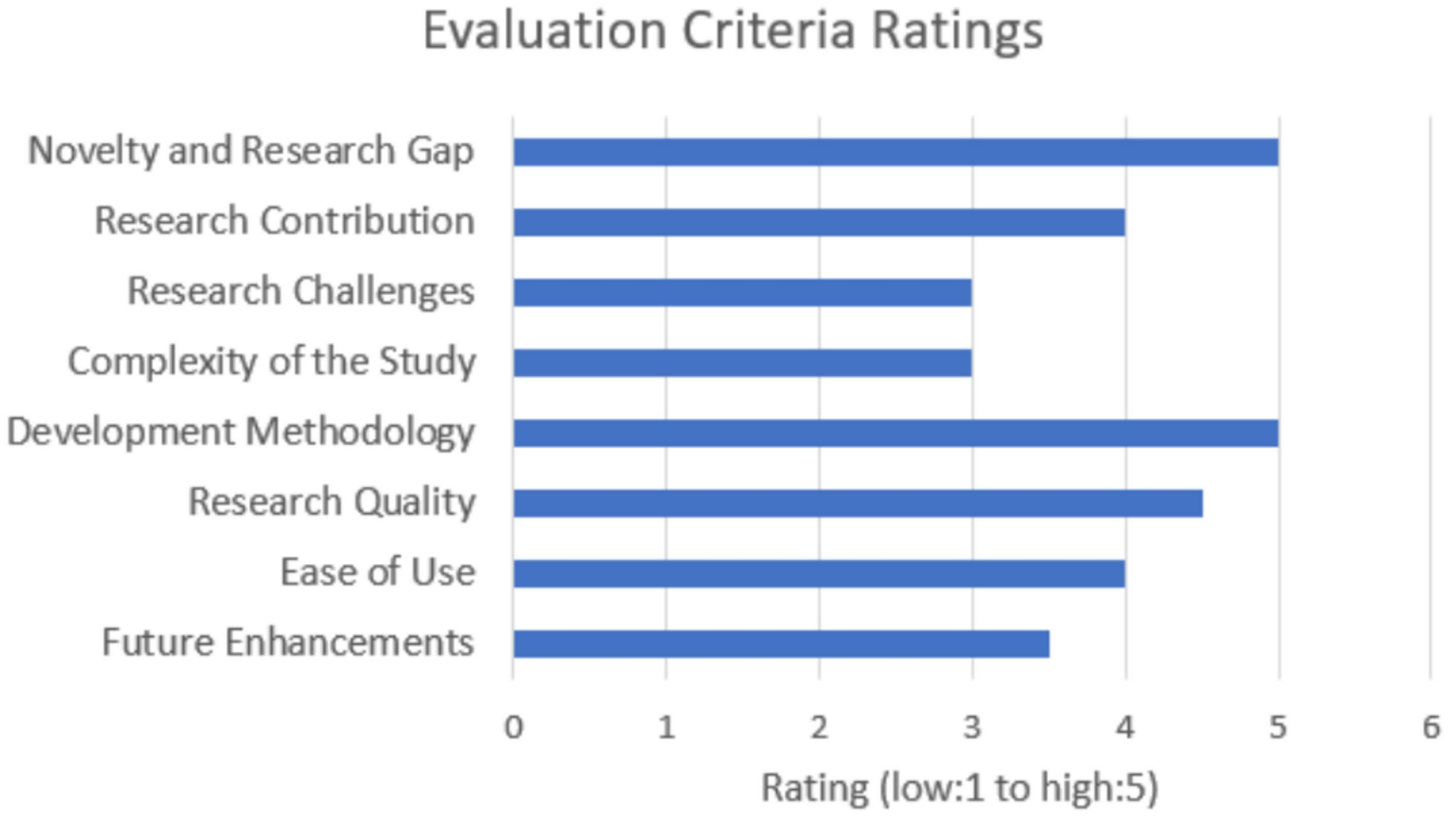
Evaluation criteria overview.

The evaluation process for this study involved engaging several experts, encompassing both domain and technical perspectives, to ensure a comprehensive assessment of the solution. The experts were provided with detailed results from the evaluation metrics, the initial design documentation, and the solution itself prior to the evaluation discussions. These 30-min discussions focused on critical criteria, including novelty and research gap, domain and project scope, research contribution, research challenges, complexity of the study, development methodology, research quality, ease of use, future enhancements, and overall feedback. This structured approach facilitated an in-depth review and constructive feedback, enabling a thorough evaluation of the research solution’s effectiveness, relevance, and potential for future improvement. The bar graph in Figure 6 denotes the average ratings for each criteria received from the evaluators.

The evaluation of the research focused on the integration of ontology-based prompt tuning into news summarization has elicited positive and insightful feedback from domain and technical experts. The evaluators widely recognized the novelty of incorporating ontology-based prompts, noting that this approach represents a significant advancement over traditional summarization models. By integrating structured domain knowledge, the research introduces a novel method for enhancing summary relevance and accuracy, which was highlighted as a major contribution to the field.

Experts commended the innovative nature of the research, emphasizing that the use of ontology-based prompts offers a substantial improvement by embedding specific domain knowledge into the summarization process. This incorporation of ontology allows for more contextually accurate and relevant summaries, addressing gaps in current models that often lack detailed domain insights. The evaluators acknowledged that this approach not only fills a critical gap but also introduces a fresh perspective on handling domain-specific information in summarization tasks.

Regarding the research’s domain and project scope, the evaluators found that the chosen domain—sports news—provided a robust framework for demonstrating the applicability and impact of the ontology-based approach. They affirmed that the project’s scope is well-defined and appropriately focused, allowing for meaningful contributions to the field. The evaluation confirmed that the integration of ontology-based prompts is practical and feasible, with the potential to advance summarization techniques significantly.

Overall, the evaluators were impressed with the research’s ability to leverage ontology-based approaches to improve summarization quality. They noted that the use of metrics such as ROGUE, BERTScore, and BARTScore provides a comprehensive assessment of the solution’s effectiveness. However, they also recommended incorporating user feedback to gain a fuller understanding of the solution’s practical performance.

In summary, the evaluators’ feedback underscores the research’s significant contributions to the field of NLP through its innovative use of ontology-based prompt tuning. They recognize the approach’s potential to enhance summarization models and address existing gaps, while also noting areas for further improvement and future enhancements to maintain its relevance and effectiveness.

## 6 Discussion

The results of our research on ontology-based prompt tuning for news summarization reveal several significant insights into the effectiveness and innovation of the approach. The findings confirm the impact of integrating domain-specific ontological knowledge into summarization models, illustrating a clear advancement in the field.

### 6.1 Discussion of the results

Our approach leverages ontology-based prompt tuning to address research gaps previously unfilled by conventional summarization methods. The inclusion of structured domain knowledge offers a notable advancement in how models handle and generate summaries. This novel integration enhances contextual relevance, a critical aspect often overlooked by traditional methods that focus primarily on surface-level patterns and generalizations. The evaluation demonstrates that our approach effectively bridges the gap by embedding detailed domain-specific knowledge, leading to more accurate and contextually relevant summaries.

The comparative analysis supports the effectiveness of our methodology with a range of metrics.

*F1 score:* Our model achieved an F1 score of *0.7639*, surpassing BART (0.7257) by *5.3%*, BERT (0.6808) by *12.2%*, GPT-3.5 Instruct (0.7267) by *5.1%*, and GPT-3.5 Turbo (0.7531) by *1.4%* highlighting the superior performance of our model compared to baseline approaches. This improvement underscores the enhanced balance between precision and recall in our summaries, confirming the efficacy of our methodology in producing high-quality, coherent content.

*ROUGE-1:* Our approach achieved an average ROUGE-1 score of *0.5828*, surpassing baseline models by approximately *5.7%* compared to GPT-3.5 Turbo (0.5207), *32.2%* compared to BERT (0.4403), and *37.9%* compared to GPT-3.5 Instruct (0.4227). This improvement indicates better term coverage and relevance in the generated summaries.

*ROUGE-2:* Our approach achieved an average ROUGE-2 score of *0.3893*, surpassing baseline models by *35.4%* compared to BART (0.2473), *80.0%* compared to BERT (0.2165), *33.3%* compared to GPT-3.5 Instruct (0.2923), and *35.5%* compared to GPT-3.5 Turbo (0.2874). This increase reflects enhanced capture of significant bigrams and multi-word phrases.

*ROUGE-L:* Our approach achieved an average ROUGE-L score of *0.4370*, surpassing baseline models by *16.5%* compared to GPT-3.5 Turbo (0.3750), *37.0%* compared to BART (0.3191), *39.0%* compared to BERT (0.3144), and *43.6%* compared to GPT-3.5 Instruct (0.3030). This result indicates superior handling of long-range dependencies and coherence.

*BARTScore:* Our model achieved an average BARTScore of *0.0495*, demonstrating a *1.2%* improvement compared to BERT (0.0494), *0.8%* compared to GPT-3.5 Instruct (0.0491), and *2.8%* compared to BART (0.0489), but is slightly lower by *2.8%* compared to GPT-3.5 Turbo (0.0509). This score confirms the effectiveness of our approach in maintaining fluency, coherence, and faithfulness in the summaries.

Implementing ontology-based prompt tuning in real-world scenarios presents several challenges, notably the continuous management of the ontology and ensuring system performance under varying conditions. Our study acknowledges these challenges and addresses them through iterative testing and modular design. For instance, system performance tests showed an average latency of 1.2 s for generating summaries, which aligns with acceptable real-time requirements but suggests potential for optimization. The results suggest that while the system performs effectively in controlled environments, ongoing evaluation and adaptation will be essential for maintaining performance and relevance in dynamic real-world applications. The modular architecture developed allows for flexibility and scalability, crucial for adapting to diverse and evolving content domains.

In summary, the results of this research validate the proposed ontology-based prompt tuning approach as a significant advancement in news summarization. The integration of domain-specific knowledge has demonstrably improved summary relevance and accuracy, addressing key research gaps and setting a new standard for future developments in the field. As the research progresses, addressing the identified challenges and exploring future enhancements will be crucial for achieving broader applicability and sustained impact.

### 6.2 Threats to validity

In evaluating the ontology-based prompt tuning approach for news summarization, different kinds of threats to validity must be taken into consideration given the fact that all experiments were done under controlled environments. These threats can impact the reliability and generalizability of our findings and are categorized into internal, external, and construct validity threats.

A significant internal validity threat is related to data quality and bias. The performance of summarization models is heavily influenced by the quality of the training and evaluation data. If the data used is biased or not fully representative of real-world scenarios, the model’s outputs may not generalize well. Despite efforts to ensure the dataset was diverse and comprehensive, inherent biases in news reporting could still affect the results. Additionally, there is a risk of model overfitting, where the model performs exceptionally well on training data but fails to generalize to new data. To mitigate this risk, cross-validation techniques and separate validation and test datasets were employed. However, overfitting remains a potential concern that could impact the model’s real-world applicability (Cao et al., 2024).

External validity threats include domain specificity and scalability. The ontology-based approach was specifically evaluated on sports news articles, and its effectiveness in other domains remains unexplored. The performance of the model in different text domains or languages has not been tested, which limits the generalizability of the findings to other contexts (Hahn and Mani, 2000). Moreover, the controlled environment in which the model was tested may not fully reflect real-world conditions. Variations in data quality and system constraints in real-world applications could lead to performance differences compared to the controlled test environment (Hahn and Mani, 2000).

Construct validity is threatened by the limitations of the metrics used for evaluation. While ROUGE, F1 Score, and BARTScore provide valuable insights, they have inherent limitations and may not capture all aspects of summary quality. For instance, these metrics might not fully address the model’s ability to capture the intended meaning or the coherence of summaries over extended. Additionally, the effectiveness of the ontology-based prompt tuning approach relies on the accuracy and completeness of the integrated ontological knowledge. Inaccurate or incomplete ontological representations could negatively impact summary quality. Ongoing updates and validation of the ontology are necessary to maintain its relevance and accuracy.

Therefore, while the ontology-based prompt tuning approach shows notable improvements in summarization performance, recognizing these threats to validity is crucial for a comprehensive understanding of the research findings. Addressing these threats through continuous research and refinement will enhance the robustness and applicability of the proposed methodology.

## 7 Conclusion and future work

The integration of domain-specific knowledge has proven to advance summarization quality, and this conclusion aims to contextualize these results while proposing directions for future enhancements.

### 7.1 Impact and relevance

This research has substantially advanced the field of news summarization by introducing a novel ontology-based prompt tuning approach. This methodology integrates structured domain knowledge into the summarization process, addressing limitations of conventional methods that often fail to capture contextual nuances. The enhanced performance of our approach, evidenced by superior evaluation metrics, highlights its ability to generate more precise and contextually relevant summaries compared to existing techniques.

The practical impact of this research is significant, particularly in environments where the accuracy and relevance of information are paramount. By setting a new benchmark for summarization quality, our methodology not only improves the fidelity of summaries but also provides a robust framework for future advancements in the field. The demonstrated improvements in metrics such as ROUGE, BERTScore, and BARTScore affirm the efficacy of our approach and its potential for widespread application.

Furthermore, the relevance of this work extends to various domains where high-quality summarization is critical, including news media, academic research, and information retrieval systems. The integration of domain-specific knowledge enhances the utility of summaries, making them more applicable and informative for end-users.

In conclusion, this research offers a significant contribution by establishing a new standard for ontology-based summarization techniques, providing both theoretical insights and practical benefits. The advancements made set the stage for future innovations and applications in the field, reinforcing the importance of context-aware summarization methodologies in addressing the evolving demands of information processing.

### 7.2 Future work

The research presented in this paper establishes a solid foundation for integrating ontology-based prompt tuning with abstractive text summarization, aimed at enhancing the accuracy and relevance of news article summaries. Despite these advancements, several promising avenues for further investigation remain.

One potential direction is the exploration of enhancing the system’s robustness and scalability by dynamically expanding the ontology knowledge base. This could involve the continuous assimilation of new information from input articles, allowing the system to adapt to emerging trends and new domains. Such a mechanism would ensure that the ontology remains comprehensive, relevant, and capable of supporting scalable summarization across various topics and contexts. By maintaining a dynamic and evolving ontology, the system can better capture new information and emerging patterns, improving the overall accuracy and effectiveness of generated summaries.

Additionally, future research could focus on further strengthening the system’s domain transferability. By developing adaptive prompt generation techniques, the model could be fine-tuned to handle different domains more effectively. These techniques would allow the summarization system to adjust to diverse content types and domains, thereby increasing its applicability across a wide range of news topics, regardless of domain-specific terminology or writing styles.

Another critical area of future exploration involves the maintenance and continuous updating of dynamic ontologies. Ensuring that the ontology evolves over time to reflect new knowledge and changing contexts is key to maintaining the relevance of the summarization process. Research into automated methods for dynamically curating and managing ontologies could offer significant advancements, enabling the system to sustain its accuracy and adaptability without manual intervention.

Together, these future research directions will not only build upon the current findings but also contribute to the advancement of more robust, scalable, and domain-flexible text summarization technologies, improving the accessibility, relevance, and utility of vast amounts of news data in a rapidly evolving information landscape.

## Data Availability

The original contributions presented in the study are included in the article/supplementary material, further inquiries can be directed to the corresponding authors.
